# When laughter arrests speech: fMRI-based evidence

**DOI:** 10.1098/rstb.2021.0182

**Published:** 2022-11-07

**Authors:** B. Westermann, M. Lotze, L. Varra, N. Versteeg, M. Domin, L. Nicolet, M. Obrist, K. Klepzig, L. Marbot, L. Lämmler, K. Fiedler, E. Wattendorf

**Affiliations:** ^1^ Department of Neurosurgery, University Hospital Basel, Basel, Switzerland; ^2^ Faculty of Science and Medicine, University of Fribourg, Fribourg, Switzerland; ^3^ Faculty of Medicine, University of Greifswald, Greifswald, Germany; ^4^ College of Health Sciences Fribourg, Fribourg, Switzerland

**Keywords:** fMRI, laughter, tickle, touch, vocalization

## Abstract

Who has not experienced that sensation of losing the power of speech owing to an involuntary bout of laughter? An investigation of this phenomenon affords an insight into the neuronal processes that underlie laughter. In our functional magnetic resonance imaging study, participants were made to laugh by tickling in a first condition; in a second one they were requested to produce vocal utterances under the provocation of laughter by tickling. This investigation reveals increased neuronal activity in the sensorimotor cortex, the anterior cingulate gyrus, the insula, the nucleus accumbens, the hypothalamus and the periaqueductal grey for both conditions, thereby replicating the results of previous studies on ticklish laughter. However, further analysis indicates the activity in the emotion-associated regions to be lower when tickling is accompanied by voluntary vocalization. Here, a typical pattern of activation is identified, including the primary sensory cortex, a ventral area of the anterior insula and the ventral tegmental field, to which belongs to the nucleus ambiguus, namely, the common effector organ for voluntary and involuntary vocalizations. During the conflictual voluntary-vocalization versus laughter experience, the laughter-triggering network appears to rely heavily on a sensory and a deep interoceptive analysis, as well as on motor effectors in the brainstem.

This article is part of the theme issue ‘Cracking the laugh code: laughter through the lens of biology, psychology and neuroscience’.

## 1. Introduction

A fit of laughter can sometimes really incapacitate us at inappropriate moments. In such a situation, the body is so much under the control of this emotional reaction that even verbal communication is barely possible. Why is this so, and which neuronal networks are involved? The aim of our functional imaging study was to identify those regions of the brain that are implicated in the competition between laughter and voluntary vocalization. By means of this approach, we hope to further characterize the neural network that is essentially involved in the control of laughter. How is such a situation distinguished from that in which laughter is unrestrainable? Is activity in the typically implicated areas of the brain suppressed, or are other regions additionally involved? Laughter is universally recognized [1] and occurs in infants long before the advent of voluntary speech, even when hearing is impaired [2]. Laughter-like vocalization in humans is differentiable from alternative forms of vocal behaviour in other primates and in rodents [3,4]. As such, laughter may involve innate mechanisms rather than learned vocal behaviour [5]. Laughter-like vocalizations are evoked not only in an emotional situation or by tickling, but also, and more frequently, in a social context in which laughter serves as a means of communication, as in humans [6,7] the great apes and rats [4,8]. While the production of speech requires the voluntary control of articulators, such as the tongue, the jaw and the soft palate, that of laughter depends solely on changes in breath control, subglottal pressure and laryngeal tension, which lead to a stereotypical pattern of respiration that is associated with rhythmic ‘ha, ha’-like sounds. Other, barely controllable physical phenomena are also evident, such as the flow of tears, sweating, blushing of the face and even an urge to urinate. The vegetative reactions that accompany non-volitional vocalizations point to the existence of processes that are largely independent of cortical control. Indeed, some investigators go so far as to describe spontaneous laughter as a subcortical reflex [9].

The analysis of the neuronal control of overt speech and laughter by functional magnetic resonance imaging (fMRI) is problematical, owing to the occurrence of unavoidable motional artefacts. Although available data pertaining to the neuronal processes that govern overt speech [10–12] and laughter [13–17] are thus scarce, they nevertheless extend and complement those derived from pathological [18,19], stimulation [20,21] and tractography studies [22]. They are of particular relevance in distinguishing between the motor control of a postulated voluntary and involuntary pathway of vocalization. While speech can be produced only when the voluntary pathway is engaged, laughter depends greatly on the involuntary one, especially when it is associated with an emotional signal stimulating mirth and is not strategically employed for communication [7,23,24]. Studies involving monkeys reveal these pathways to be largely independent of each other [25,26]. The voluntary pathway controls neurons in the brainstem, which, via the motor cortex, innervate motor effectors either directly or indirectly through the reticular formation [27,28]. The involuntarily controlled components of vocalization rely heavily on a processing of the stimulus in the limbic system, including the anterior cingulate gyrus, the insula and the hypothalamus, prior to innervation of the reticular formation and thence the motor effectors [29]. On the basis of available data in humans, a separation of these two pathways is less apparent. It has been postulated that during laughter the two pathways interact at the level of either the (pre)-supplementary motor area [7,22], the anterior cingulate gyrus [30,31] or the primary motor cortex [15,31], possibly via inhibitory or modulatory processes [7,15]. However, subcortical regions have not been discussed concerning their interaction.

By virtue of the present investigation, in which speech was interrupted by spontaneous laughter, we hope to gain further insight into the neural correlate of an interaction between the voluntary and involuntary pathways of vocalization. In this situation, the impact on the laryngeal component of vocalization is particularly notable, and necessarily involves changes in the neuronal activity of the laryngeal motor neurons, which originate from the nucleus ambiguus of the brainstem [32]. Interestingly in this context and concerning a constriction of the vocal folds, the descending cortical control of the laryngeal muscles, responsible for humming and the production of voluntary speech, is interrupted by the laryngeal-adductor-reflex (LAR) [33]. This reflex activates the nerves feeding the laryngeal adductors in response to an appropriate stimulus and prevents an inflow of air. Are there similarities between this reflex and the situation in which voluntary vocalization competes with spontaneous laughter? And is the effect of competition between the two systems manifested at the level of the effector neurons in the brainstem or at a higher stage of neuronal control? We investigated the neuronal activity of volunteers who attempted to voluntarily produce vocalizations under the provocation of laughter by a tickling stimulus. To facilitate an informative comparison within the same experimental setting, the participants were also subjected either to the tickling stimulus without being requested to voluntarily produce vocalizations or to mere touching. We also recorded the neuronal activity during the anticipation of the tickling stimulus and the influence thereon of the voluntary production of vocalizations. In contrast to previous studies, in which observations appertaining to the voluntary neural network were made independently of those relating to the involuntary one of vocalization, the aim of the present investigation was to ascertain whether the two sub-systems are interrelated.

## 2. Material and methods

### (a) Participants

Of the total 38 participants who volunteered to take part in the experiment, we included 30 (24 females and 6 males; mean age = 23.0 years; age range: 20–30 years) in the fMRI image evaluations. We excluded two individuals because of technical constraints during the process of image acquisition. Scan-to-scan frame-displacements were evaluated by summing up the data for the six head-movement parameters provided by the SPM re-alignment procedure (G motion parameters). Six participants were excluded because the number of displacements exceeding 1 were higher than a limit of 60% relative to the participant that showed the highest number of displacements exceeding 1. The validity of this approach has been verified in an earlier investigation on laughter. In this previous work the neuronal activity of the participants selected in the indicated manner failed to correlate with the head-movement parameters [16]. The informed consent of all participants was obtained, and the procedure was approved by the Ethical Committee of the University Hospital of Greifswald, Germany (BB063/10a).

### (b) Experimental design

**Figure 1.**
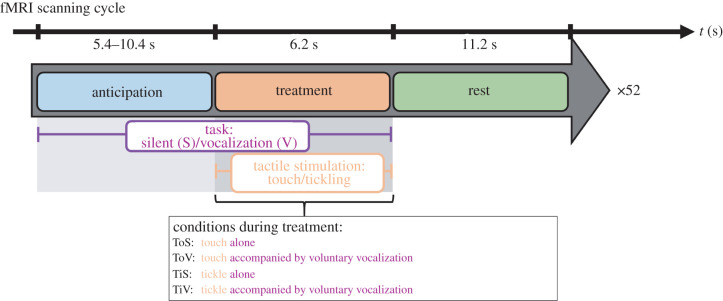
Experimental design. During the fMRI-scanning procedure, the participants repetitively experienced two different sensory stimulations, which were randomly applied during the treatment period: monotonous contact (touch) or tickling of the right foot. A visual cue signalled the upcoming stimulation, which followed after a variable delay—the phase of anticipation—of 5.4–10.4 s. A single cycle of anticipation of the stimulation, treatment and the following resting phase is depicted. At the onset of anticipation participants were encouraged to either remain silent (S) or regularly produce ‘ha’-vocalizations (V). The subjects were asked to continue the task during the treatment phase.

The fMRI investigation was based on an event-related paradigm, which included effects of anticipation (A) and stimulus application (treatment) (figure 1). At the beginning of the anticipatory period (A) participants were encouraged to either remain silent (S), or regularly produce ‘ha’-vocalizations (V). Anticipation (A) was followed by the treatment phase (T), where the participants were subjected to either tickling or a monotonous foot contact (touch) on the right foot, by a friend or partner. Silent (S) and vocalization (V) tasks began with anticipation (A) and persisted during the treatment phase (T). The onset of anticipation (A) was signalled to both the tickler and the tickled person by a smiley face appearing on the screen. While the examined participant remained uncertain about onset and type of the applied stimulus during treatment (T), a red bar, which was superimposed on either the left or the right side of the screen, informed the tickler to start the treatment (T). These settings in combination with a variable anticipatory duration (jitter) of up to 10.4 s (min. 5.4 s, max. 10.4 s) were expected to minimize habituation and to improve unpredictability of the sensory stimulation. Based on our paradigm we were able to define, for each participant, four treatment and corresponding anticipatory conditions: ToS, touch alone (12×); ToV, touch accompanied by voluntary vocalization (13×); TiS, tickle alone (13×); TiV, tickle accompanied by voluntary vocalization (14×). The number of events (shown in parentheses) varied between the different conditions because of technical issues. The investigation of the treatment phase (T) may be difficult owing to the individual's urge to move during the stimulation. To reduce movement artefacts during the fMRI image acquisition, we instructed the participants to hold a wooden barbecue stick between their teeth. This procedure prevented the jaw from opening when laughing and thus triggering a counter movement in the head. Thereby, susceptibility artefacts at air–tissue and air–bone interfaces were minimized and potential signal loss could be prevented. In a pilot study, we were able to confirm that the barbecue stick does not interfere with or restrict the participants' ability to laugh [15–17].

### (c) fMRI: acquisition of data

We performed imaging on a 3 T Scanner (VERIO, Siemens, Erlangen, Germany). The scanner was equipped with a 32-channel head coil. Functional blood oxygenation-dependent image acquisition based on a T2*-weighted echo planar imaging multiband sequence [34] (repetition time (TR) = 900 ms; echo time (TE) = 40.6 ms; flip angle = 54°; voxel size = 2.2 × 2.2 × 2.2 mm^3^; slice thickness = 2.2 mm, no gap; matrix size = 64 × 64; number of axial slices = 64; multiband factor = 6), and captured the whole brain. A further 198 phase-and-magnitude images were acquired in the same field of view using a gradient echo sequence (TR = 666 ms; TE (1) = 4.92 ms; TE (2) = 7.38 ms; flip angle = 60°; resolution = 2 × 2 × 2 mm^3^). T1-weighted structural images were obtained by using a three-dimensional magnetization-prepared rapid gradient echo (MPRAGE) sequence (TR = 1690 ms; TE = 2.52 ms; flip angle = 9°; voxel size = 0.97 × 0.97 × 1 mm^3^; matrix = 256 × 256 × 176 voxels; 176 sagittal slices). The fMRI examination was performed in a single run during which we gathered 1475 echo planar images (EPIs) per subject (total acquisition time 1327 s), with the exception of two participants, where owing to measurement issues we could validate only 1450 and 1158 volumes, respectively.

### (d) Acquisition and processing of auditory data

We recorded the full experimental procedure with an fMRI-adapted fibre optic microphone (MR confon, Magdeburg, Germany). For every subject, we obtained a single audio file of the length of 30 ± 5 min representing the total duration of the scanning period. After the experiment, Audacity^®^ software 2.3.3 was used to reduce background noise of the fMRI device and to label the events of our experimental procedure. As such it served to evaluate the vocal response of each participant during tickling. Laughter that was produced during tickling alone (TiS) and tickling with voluntary vocalization (TiV) was classified as follows: strong expiration without an audible vocalization was defined as ‘weak', bursts of laughter with only one audible vocalization as ‘middle' and bursts of laughter with two or more audible vocalizations as ‘strong' (see also [16]). This score reflects not only the frequency but also the intensity of audible laughter events. However, during TiV, participants, in addition to spontaneous laughter, also voluntarily produced ‘ha'-vocalizations. Thus, in this condition we were able to report only the strong outbursts of laughter that could be clearly identified according to our method.

### (e) fMRI: data analysis

To process the fMRI data, SPM12 software (Wellcome Department of Cognitive Neuroscience, London, UK; [11]), running on Matlab (MathWorks; Natick, MA, USA), versions R2017a and R2018a respectively, was used. Unwrapping of geometrically distorted EPIs was performed in the phase-encoding direction using a VDM (voxel displacement map) field map image representing the phase differences of the two previously acquired phase images. Each of the 1475 individual scans was realigned to the scan that displayed half-maximal displacement (as indicated by the translation or rotation parameters with the highest maximal deviation) to correct for movement artefacts. Each EPI was co-registered with the *T*_1_−weighted anatomical image. The co-registered *T*_1_−image was segmented and normalized to the Montreal Neurological Institute (MNI) template, the EPIs were resliced at 2.2 × 2.2 × 2.2 mm^3^. The resulting images were smoothed with a 6 × 6 × 6 mm^3^ Gaussian kernel filter (full-width at half maximum) to increase the signal-to-noise ratio.

### (f) fMRI: first-level analysis

To control for variance due to motions of the head, movement parameters that were estimated during the re-alignment procedure were introduced as regressors into the general linear model (GLM). The fMRI data were additionally adjusted for artefacts by a reduced weighting of motion-contaminated volumes using the RobustWLS toolbox [35]. An event-related analysis was conducted to separately identify the regions of the brain that were activated by the anticipation (A)—and the treatment (T)—stimuli in each subject. A reaction and neuronal conduction time of 400 ms after the request to effectively tickle was included in the modelling (see also [16]). Contrast images were calculated to assess the neuronal activity that was tested in the four conditions during treatment (ToS, ToV, TiS, TiV) and during anticipation. However, since the participants did not know during the anticipatory period whether they would be stimulated by tickling or by touch (see experimental design), the two of the four tested anticipatory conditions that measured silent anticipatory activity (AS) could be merged, as could the two that were related to activity during anticipation accompanied by vocalization (AV).

### (g) fMRI: second-level analysis

To investigate task-related changes in brain activity during the treatment conditions at the group level, contrast images corresponding to the four tested conditions 'touch alone' (ToS), 'touch accompanied by voluntary vocalization' (ToV), 'tickle alone' (TiS) and 'tickle accompanied by voluntary vocalization' (TiV) (figure 1) were introduced in a 2 × 2 ANOVA. Hereby, regions that were reliably activated during both tickling conditions were identified by a conjunction analysis of TiS with TiV. The same 2 × 2 ANOVA setting of analysis allowed us to separately identify and specify neuronal activity related to a provocation of laughter by tickling on the one hand (TiS) and to a production of vocal utterances under the provocation of laughter by tickling on the other hand (TiV). To this end, the neuronal activity related to the first condition was compared with the stimulation by touch only and at the same time compared with the touch accompanied by the voluntary vocalization task. The second condition was analysed by its simultaneous comparison with the touch only (ToS) and the touch accompanied by voluntary vocalization (ToV) conditions. Hence, brain regions that were typically activated in accordance with the former objective were explored by a conjunction analysis of the TiS > ToS and TiS > ToV contrasts, and the brain regions that were activated based on the latter purpose were identified by a conjunction analysis of the TiV > ToS and TiV > ToV contrasts. For simplicity, in the following sections, we will handle the first analysis as tickle > touch and voluntary vocalization, and the second one as tickle and voluntary vocalization > touch and voluntary vocalization.

To examine task-independent activation during silent anticipation (AS) and anticipation accompanied by voluntary vocalization (AV), a conjunction analysis was performed by a within-subject ANOVA with the respective merged contrasts (AS, AV). Finally, to investigate task-related changes in brain activity during AS and AV, a 1 × 4 ANOVA analysis was conducted with the corresponding non-merged contrasts (2× AS; 2× AV), and a conjunction analysis of the corresponding contrasts opposing AS and AV was performed. In all the analyses described above, we performed an analysis of the whole brain and extracted *Z*-scores from voxels that survived at a familywise error (FWE) correction threshold of *p* < 0.05.

## 3. Results

### (a) Behaviour

Out of the 30 participants, only one produced an audible response to each of the 13 tickling stimuli that were applied during the tickle alone (TiS) (total number of laughs: 0–13; median = 10, average = 8.6) as well as tickle accompanied by voluntary vocalization (TiV) (total number of laughs: 0–13; median = 2, average = 3.9) conditions. Furthermore, one participant showed no audible reaction to the stimuli, neither during TiS nor during TiV. The Kendall's tau-b correlation analysis determined a strong correlation (*r* = 0.506, *p* < 0.001) between the laughter score (total number of laughs) during tickle alone and tickle accompanied by voluntary vocalization. The correlation between strong laughter produced in both conditions was: *r* = 0.644, *p* < 0.001. The participants rated tickling as ‘pleasant' (mean score: 7.18 (s.d.: ±1.9) of 10; no significant gender differences) and as ‘rather ticklish' (mean score: 5.35 (s.d.: ±2.1) of 10; no significant gender differences). Ticklishness correlated with the laughter score (total number of laughs) during tickle alone (Kendall's tau-b: *r* = 0.315, *p* = 0.019) and during tickle accompanied by voluntary vocalization (Kendall's tau-b: *r* = 0.416, *p* = 0.003).

### (b) fMRI: treatment

#### (i) Tickle and tickle accompanied by voluntary vocalization (conjunction analysis)

**Figure 2.**
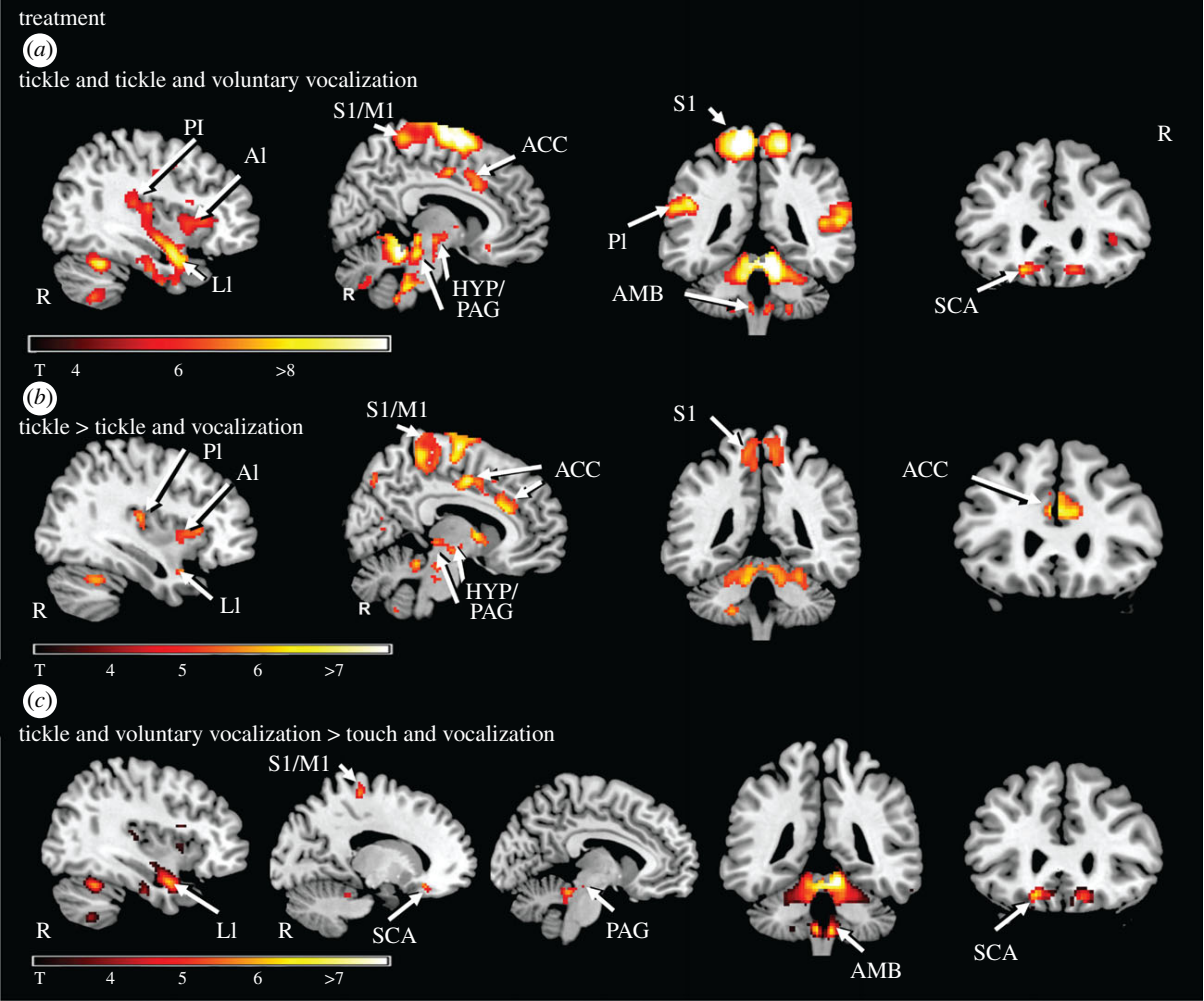
Activity during tickling and tickling in the presence of voluntary vocalization. (*a*) Conjunction analysis of tickling and tickling in the presence of voluntary vocalizations. Activation of the anterior (AI), posterior (PI) and limen (LI) parts of the insular cortex, of the primary sensorimotor cortex (S1/M1), the anterior cingulate cortex (ACC), the subcallosal area (SCA), the hypothalamus (HYP), the periaqueductal grey (PAG) and the brainstem lateral tegmental field in the area of the nucleus ambiguus (AMB) is shown. In the illustrated example, the level of significance for threshold activity was set at *p* < 0.05 (FWE-corrected). Further information concerning activation related to the nucleus accumbens and the pontine tegmentum is listed in table 1. (*b*) Brain activation during tickling compared with monotonous foot contacts. Activation of the anterior (AI), posterior (PI) and limen (LI) parts of the insular cortex, of the primary sensorimotor cortex (S1/M1), the ACC, the hypothalamus (HYP) and the periaqueductal grey (PAG) is shown. In the illustrated example, the level of significance for threshold activity was set at *p* < 0.05 (FWE-corrected). Further information concerning activation related to the middle frontal gyrus, the nucleus accumbens, the midbrain tegmentum and the anterior and the posterior cerebellar lobe is listed in table 1. (*c*) Brain activation during tickling in the presence of voluntary vocalizations compared with monotonous foot contacts. Activation of the primary sensorimotor cortex (S1/M1), the limen (LI) of the insular cortex, the subcallosal area (SCA) and the brainstem lateral tegmental field in the area of the nucleus ambiguus (AMB) is shown. In the illustrated example, the level of significance for threshold activity was set at *p* < 0.05 (FWE-corrected).

During tickling, several brain regions exhibited activation regardless of whether tickling was performed with (TiV) or without (TiS) simultaneous voluntary vocalization (table 1 and figure 2*a*). As expected [15–17], the primary sensory-motor cortex was activated, especially contralateral to the side on which the foot was stimulated. Moreover, during both tickling conditions activation was observed in posterior and anterior parts of the insular cortex and continued from the posterior regions along the long gyri to the limen insulae, which is located in the area of the anterior pole. We also replicated previous studies on tickling that reported neuronal activity in middle and anterior areas of the cingulate gyrus, in the nucleus accumbens and in the anterior and the posterior cerebellar lobes (table 1 and figure 2*a*) [15–17]. Moreover, activation of the subcallosal area (BA 25) was detected (table 1 and figure 2*a*), which in our previous studies was not differentiated from the anterior cingulate gyrus activation [15–17]. Activation of the hypothalamus and the periaqueductal grey on the other hand, confirms our earlier results (table 1 and figure 2*a*). We also detected activity bilaterally in a sharply delineated lateral region of the rostral medulla, with a peak posterior to the inferior olive. This region corresponds to the medullary lateral tegmental field and includes the nucleus ambiguus and the nucleus retroambiguus [23,36].

**Table 1.** Treatment.

anatomical brain region	hem.	cluster size (voxels)	MNI coordinates (peak)			*Z*-score^a^
			*x*	*y*	*z*	
*treatment:* *tickle and tickle and voluntary vocalization*						
primary sensory cortex	R	^b^	16	−14	70	7.39
	L	^b^	−14	36	76	8.24
primary motor cortex (incl. suppl. motor area)	R	^b^	10	−8	70	7.70
	L	^b^	−6	−8	74	7.98
dorsal anterior insula	R	^b^	46	18	−2	6.49
	L	^b^	−30	20	8	5.94
posterior insula	R	^b^	36	−18	14	6.51
	L	^b^	−36	−20	14	7.79
limen insulae (ant. inf. insular pole)	R	^b^	40	2	−22	7.43
	L	^b^	−36	0	−26	7.35
midddle/anterior cingulate gyrus	R	^b^	4	−4	44	6.30
	L	^b^	−4	−4	44	7.07
nucleus accumbens	R	133	16	6	−2	7.69
	L	66	−18	0	0	5.88
subcallosal area	R	102	10	24	−14	7.03
	L	110	−18	28	−16	7.10
hypothalamus	R	^b^	4	−16	−8	6.51
	L	^b^	−2	−16	−10	6.76
periaqueductal grey	R	^b^	8	−28	−10	6.87
	L	^b^	−6	−28	−16	6.03
tegmentum of the pontine brainstem	R	^b^	6	−36	−36	6.23
	L	^b^	−4	−36	−44	6.47
lateral tegmental field (incl. nucleus ambiguus)	R	^b^	8	−44	−52	6.23
	L	^b^	−4	−42	−50	5.60
anterior/posterior cerebellar lobes	R	^b^	34/30	−52/−58	−26/−50	7.56/7.13
	L	^b^	−32/−30	−54/−60	−28/−54	7.55/7.84
primary and secondary auditory cortex	R	^b^	48	−30	22	7.79
	L	^b^	−48	−32	22	8.02
*treatment:* *tickle* *>* *touch and voluntary vocalization*						
primary sensorimotor cortex	R	^b^	10	−8	70	6.95
	L	33	−4	−34	70	6.42
middle/anterior cingulate gyrus	R	^b^	6	−4	44	6.69
	L	^b^	−4	−6	42	6.40
middle frontal gyrus	R	158	22	50	34	5.69
	L	190	−22	−40	28	5.55
dorsal anterior insula	R	^b^	30	10	4	5.84
	L	^b^	−30	20	8	6.08
posterior insula	R	62	34	−20	14	5.73
	L	12	−34	−22	14	5.07
limen insulae (ant inf. insular pole)	R	87	36	10	−24	5.40
	L	105	−38	8	−28	5.54
nucleus accumbens	R	^b^	14	6	−2	5.97
	L	^b^	−20	8	−2	5.21
hypothalamus	R	^b^	6	−14	−10	5.81
	L	^b^	−8	−12	−4	5.34
periaqueductal grey	R	^b^	10	−26	−10	4.69
	L	^b^	−8	−26	−20	4.43
midbrain tegmentum	R/L	72	0	−24	−18	5.58
anterior/posterior cerebellar lobes	R	^b^/343	34/14	−50/−56	−30/−52	5.63/6.26
	L	^b^/219	−32/−22	−52/−46	−30/−50	5.36/6.76
*treatment:* *tickle and voluntary vocalization* *>* *touch and voluntary vocalization*						
primary sensorimotor cortex	R	48	14	−26	62	5.45
	L	67	−16	−28	64	5.60
limen insulae (ant. inf. insular pole)	R	55	38	2	−24	5.45
	L	70	−38	6	−22	6.12
subcallosal area	R	6	12	26	−16	4.93
	L	24	−16	28	−16	5.65
periaqueductal grey	R	4	10	−26	−10	4.93
	L	1	−8	−26	−14	4.72
lateral tegmental field (incl. nucleus ambiguus)	R	^b^	6	−38	−46	6.07
	L	^b^	−4	−40	−48	5.81
anterior/posterior cerebellar lobes	R	82	30	−52	−26	5.75
	L	60/19	−32/−30	−54/−60	−28/−54	5.92/5.12

^a^*Z*-scores describe the local maxima at a threshold of *p* > 0.05 (FEW).

^b^These clusters show overlapping activity with adjacent clusters.

#### (ii) Tickle > touch and voluntary vocalization

This analysis identifies neuronal activity that is present during the tickling condition in which laughter was unrestrained (TiS) compared with the touch-only condition (ToS) and compared at the same time with the touch accompanied by voluntary vocalization condition (ToV). Brain regions that were specifically involved in TiS compared with the two tested touch conditions are, with few exceptions, identical to those revealed by the conjunction analysis of the two tickle conditions, and include bilaterally the anterior and posterior regions of the insula including the limen insulae, the middle and anterior cingulate gyrus, the nucleus accumbens and the anterior and the posterior cerebellar lobes (table 1 and figure 2*b*). By contrast, no significant activation of the subcallosal area and of the lateral tegmental field in the region of the nucleus ambiguus is reported in this comparison. On the other side, activation of the midbrain tegmentum was revealed (table 1). Brain activations related to the comparison of tickle (TiS) with touch (ToS) and to the one of tickle (TiS) with touch accompanied by voluntary vocalization (ToV) are individually illustrated in electronic supplementary material, figure S1.

#### (iii) Tickle and voluntary vocalization > touch and voluntary vocalization

When laughter was provoked in the participants while they still tried to continue to voluntarily produce vocalizations (TiV) some brain regions were specifically involved compared with a stimulation by touch only (ToS) and compared at the same time with a touch accompanied by a voluntary vocalization task (ToV). In this condition, significant activity was detected in the primary sensory cortex, the limen insulae, the subcallosal area, and the anterior and the posterior cerebellar lobes in both hemispheres (table 1 and figure 2*c*). Furthermore, in the brainstem the periaqueductal grey (PAG) and the medullary lateral tegmental field were activated on both sides (table 1 and figure 2*c*). However, in contrast to the specific neuronal activity reported for the tickle condition (figure 2*b*), the one revealed for the tickling with voluntary vocalization condition was not significant in middle and anterior parts of the cingulate gyrus, in the nucleus accumbens and in the anterior and the posterior regions of the insula. Brain activations related to the comparison of tickle accompanied by voluntary vocalization (TiV) with touch (ToS) and to the one of tickle with voluntary vocalization (TiV) with touch accompanied by voluntary vocalization (ToV) are individually illustrated in electronic supplementary material, figure S2.

### (c) fMRI: anticipation

#### (i) Anticipation and anticipation accompanied by voluntary vocalization

**Figure 3.**
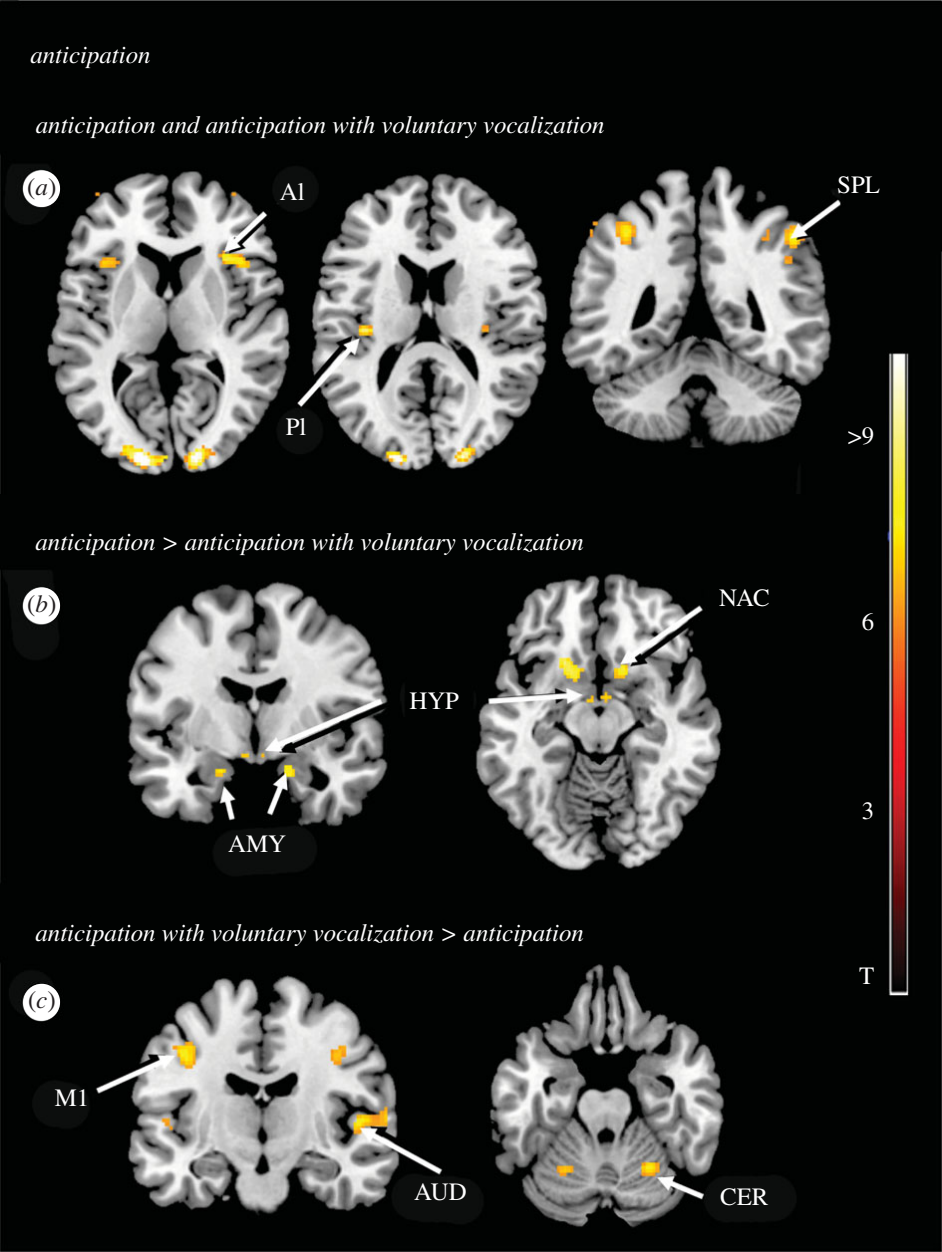
Activity during anticipation and anticipation in the presence of voluntary vocalization. (*a*) Conjunction analysis of anticipation of tickling and tickling in the presence of voluntary vocalizations. Anterior (AI) and posterior (PI) parts of the insular cortex and superior parietal lobe (SPL) are activated. In the illustrated example, the level of significance for threshold activity was set at *p* < 0.05 (FWE corrected). Further information concerning activation related to the sensorimotor cortex, the premotor cortex, the middle cingulate gyrus and the visual cortex is listed in table 2. (*b*) Brain activation during anticipation of tickling compared to anticipation of tickling in presence of voluntary vocalizations. The corpus amygdaleum (AMY), the hypothalamus (HYP) and the nucleus accumbens (NAC) are activated. In the illustrated example, the level of significance for threshold activity was set at *p* < 0.05 (FWE corrected). (*c*) Brain activation during anticipation of tickling in the presence of voluntary vocalizations compared to anticipation of tickling. The primary motor area (M1), the auditory cortex (AUD) and the anterior lobe of the cerebellum (CER) are activated. In the illustrated example, the level of significance for threshold activity was set at *p* < 0.05 (FWE corrected).

The conjunction analysis included the silent anticipation (AS) and the anticipation AV conditions to reveal the brain regions that were reliably involved in both conditions. During both anticipatory conditions (AS and AV) the sensorimotor cortex was activated primarily on the left (table 2 and figure 3*a*), which corresponds to the touch and the tickling stimulations that were applied to the right foot (see also [16]). Furthermore, expectation of both forms of the tactile stimulation activated the posterior insular cortex especially on the left, but anterior portions rather on the right side (table 2 and figure 3*a*) and evoked bilateral neuronal activity in the middle cingulate gyrus, the premotor cortex (middle frontal gyrus), the associative cortex (superior parietal lobe) and the primary and the secondary visual cortices (table 2 and figure 3*a*).

**Table 2.** Anticipation.

anatomical brain region	hemisphere	cluster size (voxels)	MNI coordinates (peak)			Z-score^a^
			*x*	*y*	*z*	
*anticipation:* s*ilent and voluntary vocalization*						
sensorimotor cortex	R	2	14	−38	64	4.93
	L	137	−8	−14	70	5.87
superior parietal lobe	R	188	50	−44	46	5.77
	L	224	−36	−50	50	6.14
dorsal anterior insular cortex	R	111	34	22	10	6.24
	L	42	−34	20	10	5.55
posterior insular cortex	R	14	32	−20	18	5.26
	L	82	−38	−20	16	6.16
premotor cortex	R	365	44	20	30	6.62
	L	11	−46	14	36	5.02
middle cingulate cortex	R	50	4	−20	32	5.85
	L					
visual cortex	R	871	18	−92	2	7.43
	L	905	−14	−94	−2	7.97
*anticipation:* *silent* *>* *voluntary vocalization*						
amygdala	R	^b^	16	12	−16	6.24
	L	24	−14	−8	−20	5.54
accumbens nucleus	R	^b^	16	12	−16	6.83
	L	^b^	−8	8	−8	5.56
hypothalamus	R	7	4	−2	−12	4.88
	L	3	−4	−4	−12	4.76
*anticipation:* *voluntary vocalization* *>* *silent*						
primary motor cortex	R	63	40	−8	38	5.34
	L	167	−44	−10	44	5.85
primary and secondary auditory cortex	R	155	50	−14	6	5.93
	L	268	−48	−18	6	7.25
anterior/posterior cerebellar lobes	R	83	26	−58	−22	5.79
	L	182	−26	−60	−20	5.9

^a^*Z*-scores describe the local maxima at a threshold of *p* > 0.05 (FEW).

^b^These clusters show overlapping activity with adjacent clusters.

#### (ii) Anticipation > anticipation accompanied by voluntary vocalization

The comparison of the condition in which the stimulation by touch or tickling was anticipated (AS) with the one in which the participants could anticipate the stimulation but at the same time had to perform a vocalization task (AV) revealed a specific involvement of several emotion-related subcortical brain regions for AS. For this condition, we were able to report augmented neuronal activity bilaterally, in the amygdala, the nucleus accumbens and the hypothalamus (table 2 and figure 3*b*).

#### (iii) Anticipation accompanied by voluntary vocalization > anticipation

The comparison of the anticipation accompanied by voluntary vocalization (AV) condition with the silent anticipation condition (AS) revealed a pattern of specific activation for AV in a few cortical regions. Here, neuronal activity was higher bilaterally in a portion of the primary motor cortex representing the control of the mouth and the larynx [15], in the auditory cortex and in the anterior cerebellar lobe (table 2 and figure 3*c*).

## 4. Discussion

The data gleaned from our analysis of tickle-induced laughter that is accompanied by voluntarily produced vocalizations accord with those of previous studies that have investigated ticklish-laughter-induced neuronal activity [15,16]. Our study reveals the involvement of the primary sensory-motor regions and the cerebellum, as well as the cortical and the subcortical areas which are considered to represent the pivotal points of the emotional motor system that are involved in the regulation of emotional vocalization. The implicated regions include the anterior and the posterior insula, the middle and anterior portions of the cingulate gyrus, the subcallosal area, the hypothalamus, the periaqueductal grey matter (PAG) and the lateral tegmental field including the area of the nucleus ambiguus [23,26,30,37]. This is the first time that the nucleus ambiguus has been implied in a laughter-eliciting task by an imaging technique. Further analysis (tickling and voluntary vocalization > touch and voluntary vocalization) demonstrated a decrease in activation of the anterior cingulate gyrus compared to tickling without the competing voluntary vocalization task. However, a significant activation in the region of the brainstem representing the nucleus ambiguus and the PAG is still registered and constitutes a component of a specific activation pattern.

### (a) Tickling with simultaneous voluntary vocalization activates visceromotor effectors in the brainstem

In this situation, neuronal activity in the lower brainstem is clearly confined to the medullary lateral tegmental field and the PAG. The former entity hosts the nucleus ambiguus, a visceromotor nucleus that directly innervates the laryngeal muscles, particularly the cricothyroid muscle which regulates the tension of the vocal folds [28], and, in so doing, has the greatest influence on the fundamental frequency of pitch [38]. The latter parameter has been shown in many studies to be a potent indicator of involuntary, emotionally-driven alterations in voice pitch [30,39,40]. The PAG is implicated in the regulation of many automatic behavioural responses [41]. It also contributes to the initiation and the intensity of any form of vocalization [42]. The control of voluntary and involuntary vocalizations converges [43], particularly at the level of the PAG and the nucleus ambiguus [44]. During involuntarily produced vocal expressions, such as laughter, motoneurons in the nucleus ambiguus are controlled by other neurons in the lateral tegmental field especially via the nucleus retroambiguus located there [23,26,31], the latter being involved in coordinating respiration with vocalization. Similarly, voluntary vocal utterances appear to be a product of phonetic patterns in the lateral tegmental field. But in contrast to involuntarily evoked ones, they are also directly attuned by modulation of the motoneurons in the nucleus ambiguus from the primary motor area [24,31]. Overall, during vocalization, neurons in the lateral tegmental field including the laryngeal motoneurons in the nucleus ambiguus integrate inputs from several regions. Augmented activity therein could thus be indicative of a parallel triggering of volitional and spontaneous vocalizations and of an engagement in the functioning of the common brainstem effector. Indeed, in the participants in whom laughter is evoked by tickling with concomitant voluntary vocalization, this response is more pronounced than when tickling was not accompanied by voluntary vocalization. Hence, in contrast to the triggering of the laryngeal adductor reflex that interrupts the production of speech production as described earlier, none of the competitive systems are completely switched off.

### (b) Tickling with simultaneous voluntary vocalization activates a somato- and viscerosensory network

The insular cortex is recognized for its role not only in the in processing of visceral stimuli but also in other emotionally arousing experiences that are physically manifested, such as the expression of disgust, fear, happiness, sadness or sexual desire, or in bodily sensations that are processed by the skin, such as changes in temperature, as well as in those related to touch and tickling [45]. The totality of the triggering stimuli has been described as interoception [46]. After pre-processing in the posterior insula [47] the information is conveyed to the anterior portion incorporating cognitive and emotional evaluations [37,48]. Here, the signal is registered consciously and is assigned to a subjective sensation [49]. In addition, adequate autonomic reactions are prepared [50,51]. In the anterior insula, two regions with different specializations are distinguishable: The so-called ‘orbital viscerosensory’ region is considered to represent bodily sensations related to an emotional experience [52]. The other region has been described as a ‘medial visceromotor' or an ‘emotional motor network', owing to its descending connections with visceromotor centers in the brainstem [53,54]. An analysis of our data reveals the two tested conditions, namely, tickling with and without voluntary vocalization to involve not only the posterior primary interoceptive cortex but also the viscerosensory and the visceromotor anterior areas of the insular cortex (compare also with [45]), the observed activity extending ventrally from the primary interoceptive cortex along the long gyri towards the viscerosensory portion of the anterior insula, close to the limen insulae. Posterior-to-anterior integration of the stimulus in the ventral areas of the insula has been described to support an awareness of the tickling stimulus along a socioemotional axis [45]. Our analysis further reveals that, unlike tickling in the absence of voluntary vocalization, tickling that is accompanied by vocal utterance specifically involves the insula only in the vicinity of the limen insulae, namely in the viscerosensory portion of the anterior insula. In contrast to the visceromotor insular cortex, this area is not characterized by extensive brainstem connections [53]. Hence, the tickling stimulus presumably triggers an emotional experience that is suppressed. For this tickling condition that competes with voluntary vocalization, the activity in the anterior cerebellar lobe points to the importance of early processing steps [55,56], whereas that in the primary sensory cortex signallizes an awareness of the topographic properties of the sensory stimulation.

### (c) Tickling with simultaneous voluntary vocalization reinforces network conflicts

Our analysis reveals activity in the subcallosal area (BA 25) during tickling with simultaneous voluntary vocalization. The medial prefrontal cortex, which harbours this cortical region, is involved in the selection of socially appropriate emotions and interactions [57]. Together with other areas of the medial prefrontal cortex, emotional and physiological responses to stimulation are regulated to render individual behaviour effective. This adjustment is important in conflictual situations that necessitate modifications in behaviour [58]. For example, the BA 25 interacts with the amygdala to inhibit responses to fearful cues [59]. Cortical and subcortical efferent projections to the agranular insular cortex, the nucleus accumbens, the mediodorsal thalamus, the posterior portions of the hypothalamus, the PAG and the ventral tegmental area have also been demonstrated [60–62]. Several previous studies revealed that these brain regions participate in the processing of stimuli that trigger laughter [15,63,64]. In the conflictual situation in which the participants willingly wish to continue vocalizations but are involuntarily brought to laughter, augmented activity in the BA 25 may aid the performance of the voluntary vocal task at the expense of the emotional response.

### (d) Tickling with simultaneous voluntary vocalization suppresses activity in the emotional network, even during the period in which tickling is only anticipated

During tickling that is accompanied by voluntary vocalization, neuronal activity that is related to the evaluation of the affective and physical qualities is involved, whereas that appertaining to the processing of the emotional dimension of the stimulus is diminished. The anticipation of sensory stimulation evokes activity in brain regions that play an essential role in its actual experience [65,66]. Hence, not surprisingly, during the anticipation of the emotional sensory experience of tickling, similar responses to those that are evoked by the actual stimulation are observed. In the former situation, activation of the primary sensorimotor cortex and the somatosensory association cortex in the superior parietal lobe indicates a concerted preparation for the sensory stimulus, irrespective of whether simultaneous voluntary vocalization occurred or not. Activity in the posterior and the anterior insula cortices augur preparation for the emotional response in accordance with the affective dimension of the stimulus during both tasks [16]. By contrast, during the anticipation of tickling in the presence of voluntary vocalization, the activity in the hypothalamus, the amygdala and the nucleus accumbens is suppressed. Previously acquired knowledge appertaining to the emotional valence of the stimulation is not drawn upon to a notable degree, even during a period of anticipation [67,68].

**Figure 4.**
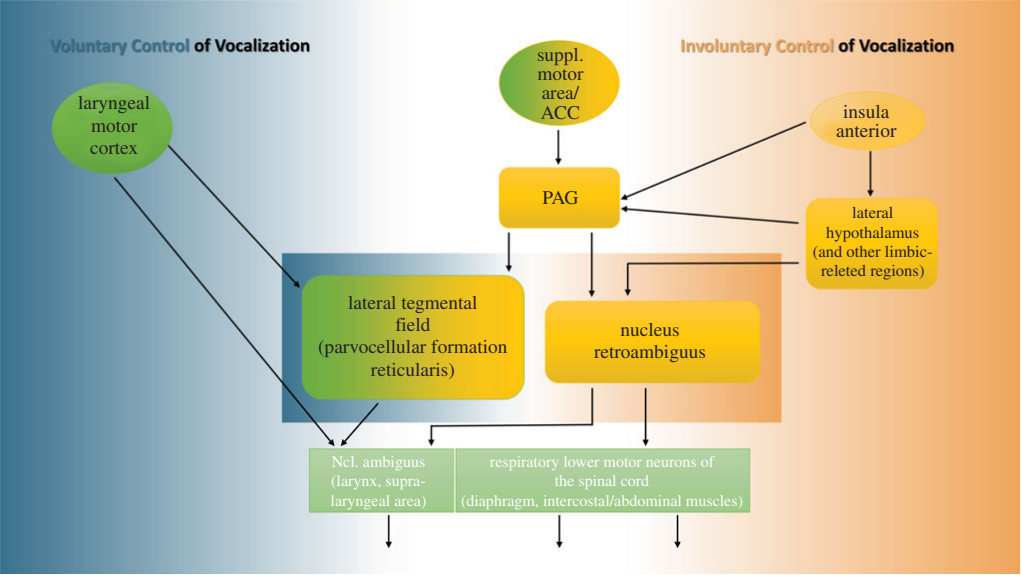
Simplified model of the efferent pathways implicated in the control of vocalization in humans (adapted from [15]). This Figure illustrates the interaction of key components of the voluntary and the involuntary pathways of vocalization. Highlighted in the centre are the regions of the brainstem that harbour the premotor interneurons and the motor neurons that control the laryngeal effectors (nucleus ambiguus) and respiratory laryngeal coordination (nucleus retroambiguus). The periaqueductal grey (PAG) is crucial for the involuntary control of vocal expression via its connections with the nucleus ambiguus and nucleus retroambiguus. Activity in the PAG is driven by that in regions associated with the limbic system, including the hypothalamus, the anterior insula and the anterior cingulate cortex (ACC). The latter is also a target of efferences of the (pre-)supplementary cortex, which is considered to bridge the voluntary and the involuntary control of vocalization [22]. The laryngeal motor cortex exerts voluntary vocal control, either directly, via connections with laryngeal effectors in the nucleus ambiguous, or indirectly, via the lateral tegmental field. Brain regions coloured in yellow represent, or bear reference to the emotional motor pathway; those in green denote the voluntary motor pathway; and those in lime green depict the effectors for vocal output.

Key components of the voluntary and the involuntary pathways that control vocalization are represented in figure 4. An interaction at different cortical and subcortical levels is possible. As far as laughter is concerned, an inhibitory or modulatory influence on the involuntary pathway involves the supplementary motor area, the cingulate gyrus and the primary motor cortex, as previously suggested [7,15,22,30,31]. In the present fMRI-investigation, which addressed the interruption of speech by laughter, we aimed to identify the regions of the brain that are implicated in the interaction between the two pathways. A verification of the interaction in the cortical regions was, however, not possible. Firstly, because the continuity of speech likewise involved continuous activity in the primary motor region, thereby obfuscating inhibitory effects thereon. And secondly, owing to a suppression of emotional processing. Notwithstanding these drawbacks, our data point to the reticular formation in the brainstem (nuclei and neuronal connections) as a possible focus of interest. As it is apparent in figure 4, this region is the point of convergence of both modes of control, which could interact in many different ways, as suggested earlier. Not least among the possibilities, would be an interaction between the premotor interneurons and the motoneurons that control the respiratory and the laryngeal effectors [69]. A further investigation of this question, however, would also require that neuronal activity in the nucleus ambiguus is being distinguished from that of other neuronal assemblies surrounding it. Moreover, we may have failed to capture all of the effects of the emotional stimulation in this study, which can be manifested also in much subtler ways. An influence of the emotional stimulation may become apparent, without interrupting the volitionally produced ‘ha, ha'-sounds, as a change in pitch [38]. In this situation, although the voluntary respiratory control would still be intact, the stimulation would impact only the laryngeal effectors. In a future analysis, these changes in pitch could be recorded, and would represent the associated brain activity.

## 5. Conclusion

By using a tickling/speech performance test in conjunction with the recording of neuronal activity by fMRI, we have experimentally demonstrated how involuntary laughter and the control of speech interact on a neuronal basis. In this competitive situation, augmented neuronal activity is recorded in the lateral tegmental field including the area of the nucleus ambiguus nucleus that controls the larynx, i.e. the common effector organ for any form of vocalization. Sensory regions that analyse the different modalities of the stimulus are also implicated. On the other hand, neuronal activity is suppressed in the cortical centers that elicit an emotional response. In this situation, the activated network suffices to trigger laughter, albeit to a lesser degree.

## Data Availability

Data underlying the final statistics of the results can be found at https://github.com/NitramNimod/When-laughter-arrests-speech-fMRI-based-evidence. Electronic supplementary material is available online [70].
